# Anxiety in Hypothyroidism: High Prevalence and Significant Treatment Response in a Rural Nepalese Cohort

**DOI:** 10.7759/cureus.92508

**Published:** 2025-09-17

**Authors:** Merina Pandey, Narayan Prasad Neupane, Bhabiswar Tiwari, Bibek Aryal

**Affiliations:** 1 Medicine, Shringa Primary Healthcare Centre, Gulmi, NPL; 2 Medicine, MultiCare Tacoma General Hospital, Tacoma, USA; 3 Surgery, Grande City Hospital, Kathmandu, NPL

**Keywords:** anxiety, hamilton anxiety rating scale, hypothyroidism, iodine, levothyroxine

## Abstract

Objectives: The association between hypothyroidism and anxiety symptoms is well-established. However, there is limited research on this comorbidity in resource-constrained rural settings, such as Nepal, where delayed diagnosis may exacerbate psychiatric burden. This study aimed to assess the prevalence of anxiety among hypothyroid patients in rural Nepal and evaluate the impact of levothyroxine therapy on their symptoms. A key secondary objective was to explore the association between dietary iodine intake and residual post-treatment anxiety.

Methods: This cross-sectional study included 101 hypothyroid patients (mean age: 53.7±1.4 years; 88 (86.3%) female) receiving levothyroxine therapy (median dose: 37.5 mcg (IQR 25-62.5); median duration: 5 years) at a primary health center in Shringa, Nepal. Anxiety severity was assessed using the Hamilton Anxiety Rating Scale (HAM-A) before and after levothyroxine treatment. Dietary iodine intake was also evaluated.

Results: Post-treatment HAM-A scores were significantly lower than pre-treatment scores, with a median decrease of 14 points (P<0.0001; Wilcoxon matched-pairs signed-rank test). Severe anxiety (HAM-A ≥19) was present in 55 (54.5%) patients, moderate in 19 (18.8%) patients, mild in 14 (13.9%) patients, and none in 13 (12.9%) patients. Mean daily salt intake was 10.47±2.57 g, and median daily iodine intake was 340 mcg (IQR 306-408). Dietary iodine intake correlated positively with post-treatment HAM-A scores (*r*=0.23, P=0.02).

Conclusions: Anxiety is highly prevalent among hypothyroid patients in this rural Nepalese setting. Levothyroxine therapy substantially alleviates these anxiety symptoms. For practitioners in rural and remote settings, these findings underscore the importance of managing the psychiatric dimensions of hypothyroidism. Integrating anxiety assessment into routine hypothyroidism care is essential for improving patient outcomes in rural settings.

## Introduction

Hypothyroidism is frequently associated with a range of neuropsychiatric manifestations, with the link between thyroid dysfunction and anxiety symptoms being well-established in both clinical practice and research [1-5]. The association between hypothyroidism and anxiety underscores the importance of recognizing and managing the psychological dimensions of thyroid disease to improve patient outcomes. While the prevalence and characteristics of hypothyroidism have been explored in various populations, the nuances of its presentation and the impact of treatment can differ based on sociodemographic factors and healthcare accessibility, particularly in rural regions with limited medical resources.

Despite the established association between hypothyroidism and anxiety, there is limited research on this comorbidity within resource-constrained rural settings. In places such as rural Nepal, the paucity of information is critical, as factors like delayed diagnosis and restricted access to mental health services can amplify psychiatric manifestations. The effectiveness of treatment in these settings also remains insufficiently understood. Therefore, this study aimed to investigate the prevalence of anxiety symptoms among hypothyroid patients in a rural Nepalese population and to assess the impact of levothyroxine treatment on these symptoms.

Further contextualizing thyroid health in the region, Nepal initiated a universal salt iodization program (starting with a five-year plan from 1998-2003) to combat iodine deficiency, with the Nepal Food Act specifying iodine levels in salt (at least 50 ppm at packaging and at least 30 ppm at retail) [6]. However, observations indicate excessive iodine concentration in household salt, prompting timely attention to iodized salt status [7]. Given that imbalances in iodine intake, both deficiency and excess, can impact thyroid function and, consequently, neuropsychiatric health, this study also evaluated dietary iodine consumption to investigate its potential association with the anxiety symptoms observed in our cohort.

## Materials and methods

Study design and participants

The study was conducted in a typical rural primary care setting for the region, characterized by limited access to consistent laboratory services, no on-site specialist care (such as endocrinology or psychiatry), and reliance on paper-based medical records. This cross-sectional study was conducted from January 7 to January 15, 2025, in Chhatrakot Municipality of Gulmi, Nepal. A total of 101 patients with clinically diagnosed hypothyroidism were enrolled. Participants were recruited from six different wards with the assistance of local Female Community Health Volunteers (FCHVs).

Ethics approval and consent to participate

This study was conducted in full accordance with the principles of the Declaration of Helsinki. The research protocol, informed consent forms, and all related study documents received ethical approval from the Nepal Health Research Council (NHRC) through an expedited review process (Protocol Registration No: 629_2024). Prior to enrollment, all participants were provided with a detailed explanation of the study's objectives and procedures. Written informed consent was subsequently obtained from every participant. Participation was entirely voluntary, and patients incurred no additional costs for their involvement. All personal data were anonymized to protect participant privacy, and access to the data was strictly restricted to authorized research personnel. The study was officially classified as posing minimal risk to participants by the NHRC.

Inclusion criteria

Participants had to be 18 years of age or older to be included in the study. Patients were required to provide recent outpatient medical records to confirm their diagnosis and treatment status. As acknowledged in our limitations, baseline diagnostic laboratory data were often unavailable in the historical records in this setting. Therefore, for the purpose of this study, "confirmation of diagnosis" was based on a review of the patient's outpatient medical records, which had to contain: (1) a documented clinical history consistent with hypothyroidism, and (2) an active, ongoing prescription for levothyroxine. We recorded the current medication dose and the approximate start date to verify active treatment status and duration.

Exclusion criteria

Individuals were excluded if they were not currently under treatment for hypothyroidism, either because it had not yet started or had been discontinued.

Data collection and procedures

All eligible participants were interviewed to collect demographic data and information on medical comorbidities. Neuropsychiatric symptoms were evaluated using the Hamilton Anxiety Rating Scale (HAM-A). The scale was administered to assess two distinct time points: current anxiety symptoms while on treatment, and a retrospective assessment of anxiety symptoms experienced before the initiation of levothyroxine therapy. Given that the median duration of treatment was five years, this retrospective assessment is subject to a long recall period, which may impact the accuracy of the baseline anxiety data.

To estimate daily dietary salt and iodine intake, a pictorial diagram of tablespoon measurements was used to help participants quantify their salt consumption, which was subsequently converted to grams. This post-treatment intake value was the one used in the correlation analysis. 

Assessments and analyses

Hamilton Anxiety Rating Scale

The HAM-A is a well-validated, 14-item clinician-rated instrument used to quantify the severity of anxiety [8]. It assesses both psychic symptoms (such as mood, tension, and fears) and somatic symptoms, making it a reliable tool for tracking changes in anxiety in response to treatment. All assessments were conducted by qualified physicians (co-authors of this study) trained in its administration.

Iodine Content Analysis

To determine the iodine content in the salt consumed by the study population, eight salt samples were collected from a diverse selection of participants' homes across the study area. The selection of these samples was based on convenience sampling from households across different wards in the study area. This approach was chosen based on the observation that most households consume salt from the same government-subsidized brand. Our intent with these limited samples was to conduct a preliminary assessment of iodine concentration and explore potential variations, for instance due to storage, rather than to establish a statistically representative measure for the entire population. The samples were then sent to the Water Engineering and Training Centre, Nepal, to check for iodine content on a dry weight basis.

Statistical analyses

Descriptive statistics were used to summarize participants’ demographic and clinical characteristics. Continuous variables, such as age, levothyroxine dose, duration of treatment, daily salt intake, and daily iodine intake, are presented as means ± standard deviation (SD) or medians with interquartile range (IQR), as appropriate based on data distribution. Categorical variables, including sex and psychiatric medication use, are presented as frequencies and percentages.

Anxiety symptom severity was assessed using the HAM-A scale. Pre-treatment HAM-A scores were categorized into no anxiety (0-7), mild (8-13), moderate (14-18), and severe (≥19) categories, and their frequencies were reported. To evaluate the change in anxiety symptoms following levothyroxine treatment, a Wilcoxon matched-pairs signed-rank test was performed to compare pre- and post-treatment HAM-A scores.

Spearman's rank correlation analysis was used to assess the relationship between daily iodine consumption and post-treatment HAM-A scores. A two-tailed P-value of <0.05 was considered statistically significant for all analyses. Data were analyzed using SPSS Statistics, version 29.0 (IBM Corp., Armonk, NY, USA).

## Results

Table 1 presents the demographic and clinical characteristics of the study participants (n=101). The mean age was 53.7±1.4 years (SE), and the majority were female (88 (86.3%)). Most participants were farmers (97 (96%)), with a small percentage being teachers (4 (4%)). All patients were receiving levothyroxine therapy. The median levothyroxine dose was 37.5 mcg (IQR: 25-62.5), and the median duration of levothyroxine intake was five years (IQR: 2-7). Regarding psychiatric medication use, 76 (75.3%) of participants reported not taking any psychiatric medication. Among those who were treated, 16 (15.8%) used amitriptyline, three (3%) used escitalopram, three (3%) used olanzapine, and one (1%) each used quetiapine, duloxetine, and sertraline. The median duration of psychiatric medication intake was six years (IQR: 2.5-13). The mean daily salt intake was 10.47±2.57 g (SE), and the median daily iodine intake was 340 mcg (IQR: 306-408). Over half of participants (52 (51.5%)) perceived that they consumed an excessive amount of salt or followed a salty diet.

**Table 1:** Demographic and clinical characteristics (n=101) SE: standard error; SD: standard deviation; IQR: interquartile range

	Mean±SE or n (%) median (IQR)
Age, years	53.7±1.4 (SE)
Female, sex	88 (86.3%)
Occupation	
Farmer	97 (96%)
Teacher	4 (4%)
Levothyroxine dose, mcg	37.5 (25-62.5)
Duration of levothyroxine intake, years	5 (2-7)
Psychiatric medication	
Amitriptyline	16 (15.8%)
Escitalopram	3 (3%)
Olanzapine	3 (3%)
Quetiapine	1 (1%)
Duloxetine	1 (1%)
Sertraline	1 (1%)
Duration of psychiatric medication intake, years	6 (2.5-13)
Daily salt intake, g	10.47±2.57
Daily iodine intake, mcg	340 (306-408)
Perception of excess salt intake, yes	52 (51.5%)

Baseline anxiety severity (Hamilton Anxiety Rating Scale) before levothyroxine treatment

The severity of anxiety symptoms before initiation of levothyroxine therapy was assessed using the HAM-A and categorized into no anxiety (0-7), mild (8-13), moderate (14-18), and severe (≥19) (Table 2). Among the 101 patients, 13 (12.9%) had no anxiety, 14 (13.9%) had mild anxiety, 19 (18.8%) had moderate anxiety, and 55 (54.5%) had severe anxiety. This indicates that more than half of the participants experienced severe anxiety symptoms prior to starting levothyroxine therapy.

**Table 2:** Distribution of anxiety severity based on HAM-A scores before initiation of levothyroxine HAM-A: Hamilton Anxiety Rating Scale

HAM-A category	Number (%)
No anxiety (HAM-A 0-7)	13 (12.9%)
Mild anxiety (HAM-A 8-13)	14 (13.9%)
Moderate anxiety (HAM-A 14-18)	19 (18.8%)
Severe anxiety (HAM-A ≥19)	55 (54.5%)

Change in anxiety severity following levothyroxine initiation

**Figure 1:**
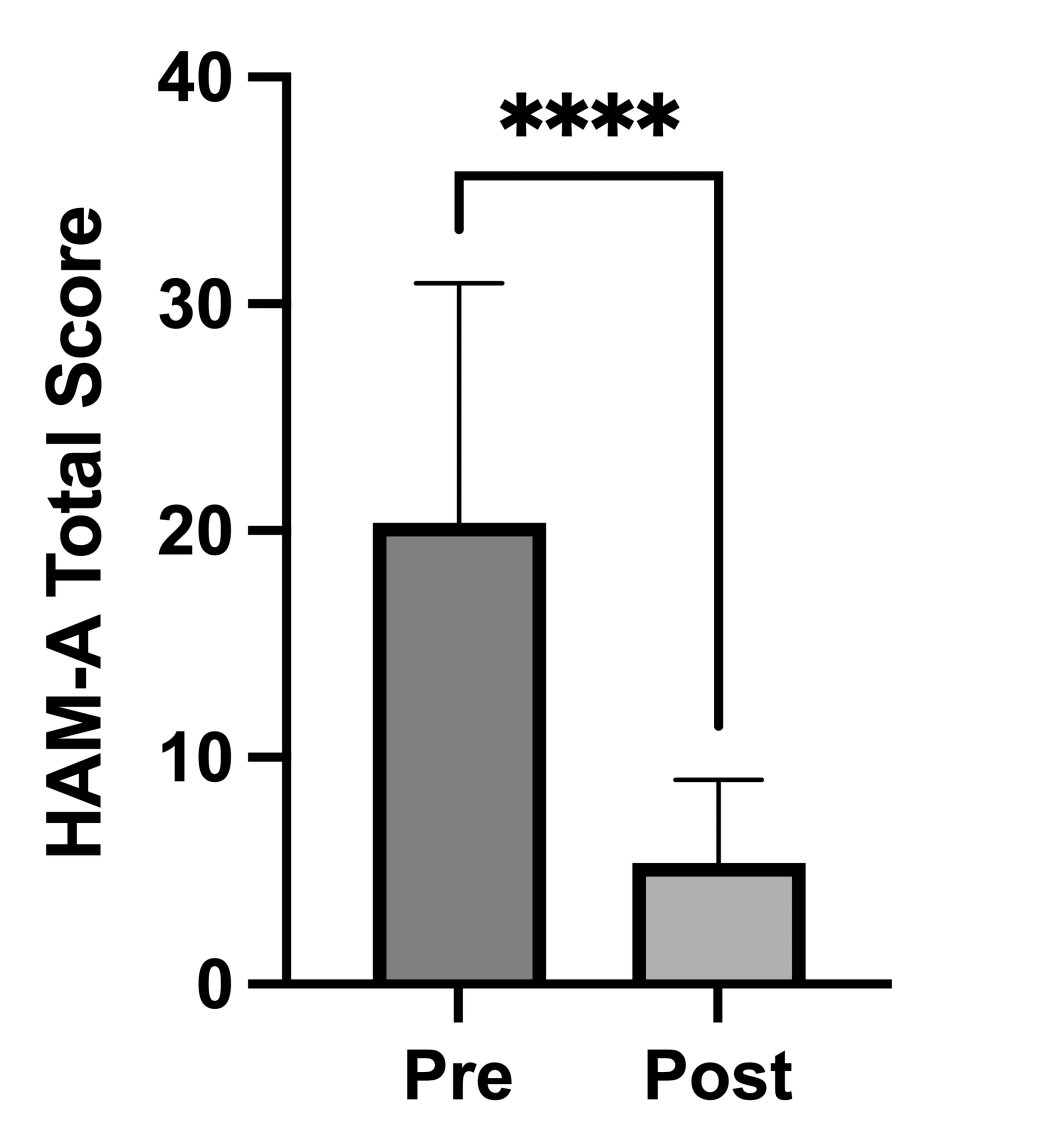
Change in HAM-A scores before and after levothyroxine treatment ^****^P<0.0001 (Wilcoxon matched-pairs signed-rank test). The figure compares the total scores on the HAM-A before (Pre) and after (Post) the initiation of levothyroxine treatment. The Y-axis represents the total HAM-A score. The bars show a statistically significant decrease in anxiety symptoms post-treatment. HAM-A: Hamilton Anxiety Rating Scale

A Wilcoxon matched-pairs signed-rank test revealed a significant reduction in anxiety symptoms after initiation of levothyroxine. Post-treatment HAM-A scores were significantly lower than pre-treatment scores, with a median decrease of 14 points (P<0.0001), indicating a robust reduction in anxiety symptoms (Figure 1).

Correlation between iodine consumption and post-treatment anxiety

**Figure 2:**
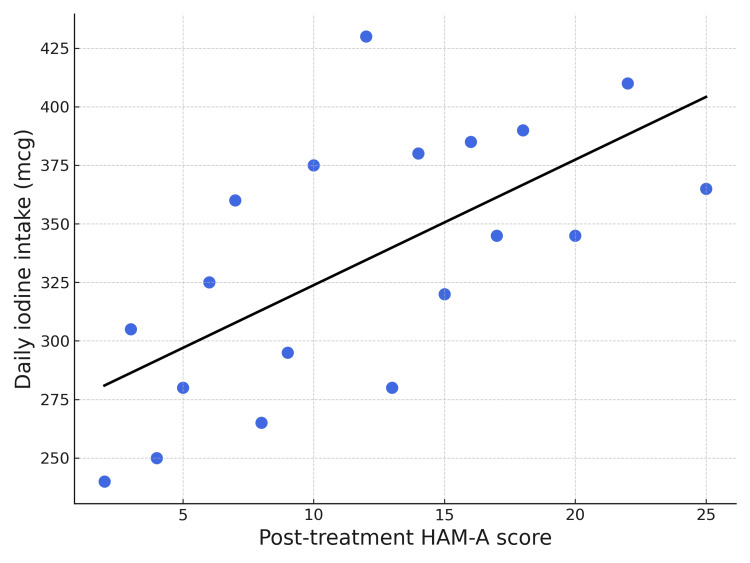
Correlation between iodine consumption and post-treatment anxiety The scatterplot illustrates the relationship between daily iodine consumption in micrograms (Y-axis) and the HAM-A scores after levothyroxine therapy (X-axis). Each point represents an individual study participant. HAM-A: Hamilton Anxiety Rating Scale

Spearman’s rank correlation analysis demonstrated a positive correlation between daily iodine intake and post-treatment HAM-A scores (r=0.23; 95% CI: 0.03 to 0.41; P=0.02) (Figure 2). This suggests that higher iodine consumption was modestly associated with residual anxiety symptoms after levothyroxine therapy.

## Discussion

This study provides a focused evaluation of anxiety disorders in patients with hypothyroidism within a specific, rural Nepalese population receiving care at a resource-constrained primary healthcare center. Our findings quantify a high burden of anxiety among these individuals prior to thyroid-specific treatment, with 55 (54.5%) patients experiencing severe anxiety symptoms. The investigation clearly demonstrates the therapeutic efficacy of levothyroxine, as evidenced by a significant reduction in anxiety severity following its initiation. A noteworthy finding was the modest positive correlation identified between higher daily dietary iodine consumption and increased residual anxiety symptoms after levothyroxine treatment, suggesting a complex interplay that warrants further exploration.

A key finding of this study is the high burden of anxiety among hypothyroid patients in rural Nepal, with 55 (54.5%) experiencing severe symptoms before starting levothyroxine. This prevalence is consistent with reports from other countries, such as Iran (58%) and India (63%) [3,9]. Similarly, studies from tertiary hospitals in Nepal have found anxiety rates ranging from 50% to 65% among hypothyroid patients [10]. However, research focusing specifically on psychiatric comorbidities in rural Nepalese populations remains limited, which this study addresses. Understanding this high prevalence requires examining both the physiological and contextual factors that may influence anxiety severity in these settings.

The high prevalence highlights the significant anxiety associated with hypothyroidism, particularly in resource-constrained rural settings where mental health services may be limited. While the direct physiological effects of thyroid hormone deficiency on mood and anxiety regulation are well-established, the severity seen in this cohort may be exacerbated by common factors such as a lack of public awareness regarding the symptoms of hypothyroidism, issues with medication availability, and financial constraints, all of which can contribute to significant delays in diagnosis and treatment initiation, thereby prolonging psychological distress [11-13]. Further comparison of these prevalence rates with those from similar resource-limited settings internationally, as well as among different demographic groups within rural Nepal (e.g., the Himalayas), would help contextualize the unique challenges faced by this population.

The therapeutic action of levothyroxine involves restoring normal thyroid hormone levels, which helps correct physiological disturbances, such as altered neurotransmitter metabolism and cerebral function, that contribute to mood and anxiety disorders in hypothyroid states [5,14]. In this study, we observed a significant reduction in anxiety symptoms following levothyroxine initiation, evidenced by a median decrease of 14 points on the HAM-A scale (P<0.0001). Although longitudinal studies quantifying HAM-A score changes with levothyroxine in hypothyroid patients are limited, prior research, including Bathla et al. [3], confirms high baseline anxiety in hypothyroid patients using the HAM-A scale. Moreover, restoration of euthyroidism is widely reported to improve psychiatric comorbidities [15,16]. Mechanistic studies, such as Liu et al., demonstrate levothyroxine-induced normalization of brain gamma-Aminobutyric acid (GABA)+ levels, providing a neurobiological basis for its anxiolytic effects [17]. Our findings thus add to the evidence supporting levothyroxine’s significant role in improving mental well-being, particularly anxiety in hypothyroid individuals.

A noteworthy finding from our study is the modest but significant positive correlation between daily iodine intake and post-treatment HAM-A scores (r=0.23, P=0.02). This suggests that higher iodine consumption was associated with greater residual anxiety, even in patients receiving levothyroxine therapy. This finding is particularly important as it raises questions about whether excessive iodine might impact the management of neuropsychiatric symptoms in hypothyroid patients. This context is critical in Nepal, where a successful Universal Salt Iodization program has been in place since 1998 [7]. While such programs are vital, some studies show a subsequent increase in thyroid dysfunction. For instance, a program in Denmark was followed by a rise in hypothyroidism [18]. Our finding, therefore, may shed light on a complex interplay between iodine intake, thyroid management, and mental health outcomes that warrants further investigation.

In this study conducted in rural Nepal, where most patients preferred a salty diet, the mean intake of salt in hypothyroid patients reached 10 grams daily. The calculated median daily iodine intake reached 340 micrograms, significantly exceeding the recommended intake of 150 micrograms. Our study also demonstrates a modest but significant positive correlation (r=0.23, P=0.02) between dietary iodine intake and post-treatment HAM-A scores. This finding raises questions about whether excessive dietary iodine also impacts hypothyroidism treatment. Our current findings align with another study by Giri et al. that highlights high iodine content in Nepalese salt [7]. These findings demand early attention from policymakers, increased vigilance, and timely changes to the iodine supplementation programs, which are long overdue in Nepal.

This study has several important limitations that should be acknowledged, many of which are inherent to its retrospective design and the setting in a rural primary care center in Nepal.

First, the retrospective design meant that baseline and follow-up biochemical data, such as TSH and free T4 levels, were unavailable. This prevents the biochemical confirmation of initial diagnoses, the differentiation between overt and subclinical hypothyroidism, and the confirmation of euthyroid status post-treatment. Furthermore, the absence of a non-hypothyroid control group from the same population makes it difficult to determine if the high anxiety prevalence is specific to hypothyroidism or reflective of broader regional challenges.

Second, our assessments are susceptible to significant recall bias. Pre-treatment anxiety scores were based on participants' memory of symptoms from several years prior, and the retrospective use of the HAM-A scale-a tool designed for cross-sectional assessment, is a major methodological weakness. Similarly, the assessment of dietary iodine was based on self-reported salt consumption rather than objective measures like urinary iodine concentration. Our design also prevented us from assessing baseline iodine status or tracking changes in salt intake after diagnosis.

Third, our study did not control for key confounding variables. We did not systematically screen for other potential causes of anxiety, such as primary anxiety disorders or comorbid substance use. Additionally, for the subset of patients on psychiatric medication, we could not ascertain the specific clinical indication for these prescriptions. Therefore, it is difficult to definitively attribute the observed anxiety solely to hypothyroidism or the improvement solely to levothyroxine.

Despite these limitations, this study provides valuable preliminary insights into the significant burden of anxiety among hypothyroid individuals in an under-researched community and underscores the need for future prospective studies to confirm and expand upon these findings.

## Conclusions

Our study reveals a high burden of anxiety among hypothyroid individuals in a resource-limited rural setting and demonstrates that levothyroxine therapy significantly reduces these symptoms. This is particularly relevant for rural healthcare providers who often manage hypothyroidism without immediate access to specialist mental health services. Furthermore, our exploratory finding of a modest association between higher iodine intake and residual anxiety post-treatment should be interpreted with caution. This preliminary observation suggests a complex interplay that requires rigorous validation in future prospective studies before any definitive conclusions or implications for public health can be drawn. To build upon our preliminary findings, we strongly recommend future prospective studies that incorporate biochemical confirmation of both thyroid function (TSH, FT4) and iodine status (e.g., urinary iodine concentration). Such robust designs are essential to definitively clarify the complex interplay between thyroid health, iodine intake, and psychiatric well-being in this population.
